# Time-Series-Based Leakage Detection Using Multiple Pressure Sensors in Water Distribution Systems

**DOI:** 10.3390/s19143070

**Published:** 2019-07-11

**Authors:** Yu Shao, Xin Li, Tuqiao Zhang, Shipeng Chu, Xiaowei Liu

**Affiliations:** 1 Department of Civil Engineering, Zhejiang University, Hangzhou 310058, China; 2 Institute of Water Resources & Ocean Engineering, Ocean College, Zhejiang University, Hangzhou 310058, China

**Keywords:** leak detection, water distribution system, data interpretation, time series, hydraulic models

## Abstract

Leak detection is nowadays an important task for water utilities as leakages in water distribution systems (WDS) increase economic costs significantly and create water resource shortages. Monitoring data such as pressure and flow rate of WDS fluctuate with time. Diagnosis based on time series monitoring data is thought to be more convincing than one-time point data. In this paper, a threshold selection method for the correlation coefficient based on time series data is proposed based on leak scenario falsification, to explore the advantages of data interpretation based on time series for leak detection. The approach utilizes temporal varying correlation between data from multiple pressure sensors, updates the threshold values over time, and scans multiple times for a scanning time window. The effect of scanning time window length on threshold selection is also tested. The performance of the proposed method is tested on a real, full-scale water distribution network using synthetic data, considering the uncertainty of demand and leak flow rates, sensor noise, and so forth. The case study shows that the scanning time window length of 3–6 achieves better performance; the potential of the method for leak detection performance improvement is confirmed, though affected by many factors such as modeling and measurement uncertainties.

## 1. Introduction

The urban water supply network system can be seen as a mobile data carrier which transmits the information of pipe flow rate, nodal pressure, and water quality under the condition of satisfying energy balance, pressure balance, and water quality balance. Being the city’s primary infrastructure, the system provides security for urban development and residential life, which requires a guarantee of the system’s safe, efficient, and economical operation. As all water distribution systems (WDS) have a certain degree of leakage, leakage management of pipe networks becomes one of the most concerning problems for water supply companies.

Over the past few decades, several researchers have conducted extensive research on leak detection in water distribution systems; a number of methods have been proposed to identify pipe bursts/leaks [1]. Leak detection techniques can be divided into two categories: external and internal [2]. External methods include radar detection, hydrophone, and acoustic logging, and are time-consuming and can only be searched locally. Internal methods are divided into four main categories: (1) transient-based methods, (2) calibration-based approaches, (3) data-driven methods, and (4) model-based techniques.

Transient-based methods determine the location of leaks through time domain or frequency domain analysis of pressure signal observations [3]. Time domain methods were introduced by Jönsson and Larson [4] and have since been derived in a number of techniques [5,6,7,8]. It consists of time domain reflection techniques, impulse response techniques, and inverse transient analysis (ITA) methods. Gong et al. [9] utilized frequency response diagrams for pipeline leak detection; Colombo et al. [10] reviewed these types of methods. However, water distribution systems are usually complex pipe networks with looped and branching topology, and contain many other components, any one of which may lead to severe attenuation of the transient phenomenon. In addition to attenuation, the method has difficulty distinguishing the transient wave responses caused by leaks from responses caused by the change of water demand and pipe fittings.

Calibration-based approaches are based on the inverse problem of parameter identification, comparing the checked model parameters with historical values, and judging whether there is a leak in the water network according to the change of parameters. Examples include leak modeling based on flow ejector principles [11], water distribution model calibration based on genetic algorithms [12,13], and pressure-dependent leak detection (PDLD) [14,15]. The disadvantage of these proposed methods is that evolutionary algorithms require a large number of solution evaluations and large-scale decision variables; meanwhile, the algorithms may fall into premature convergence easily, which further affects the accuracy of model calibration.

Extracting valid information from a large number of data samples to construct data-driven models is the core principle of the data-driven methods [3]. Traditional methods based on experiential learning theory, such as artificial neural networks (ANN) [16,17] and Bayesian inference methods [18,19], are usually based on statistical analysis. However, the ANN methods have some drawbacks, such as (1) requiring the user’s experience and prior knowledge in order to produce a good network configuration, and (2) needing sufficient training samples to achieve adequate accuracy. Meanwhile, the ANN training time increases as the size of the training samples increases. Although the Bayes methods avoid the unavoidable modeling and measurement uncertainties, the recognition accuracy is usually very low when the assumed probability distribution is different from the real probability distribution. Besides, it is difficult to accurately estimate the variance matrix. Only when the number of training samples tends to be large enough can these methods achieve better results. In order to solve the small sample problem, a machine learning method based on statistical learning theory such as support vector machine (SVM) is established [20,21,22].

Different from the calibration-based approaches, the model-based techniques consider the model to be calibrated and then establish correlations between the measured data provided by the monitoring points and the model parameters. Perez et al. [23,24] considered various uncertainties—the leakage location is carried out by comparing the threshold with the pressure residuals and combining with the leakage sensitivity matrix. The threshold is a value; when the indicator is greater than it, the leakage is considered to occur and then an alarm is issued. Casillas et al. [25] proposed a model-based leak location approach based on a new representation called the Leak Signature Space (LSS). Based on error-domain model falsification, Goulet et al. [26] and Moser et al. [27] used explicit representation of the uncertainty distribution of modeling and measurement at each location. The threshold limit for falsifying model instances in error-domain modeling is calculated to determine the candidate leak nodes. Meseguer et al. [28] proposed a model-driven decision support system which integrates the model-based leak location method based on the use of online telemetry information and a water network calibrated hydraulic model. The data and model combination methods based on Kalman filter principle [29,30,31] have the advantages of high computational efficiency, fast detection speed, and no requirement for a large amount of training data.

Most of the existing model-based or data-driven methods mentioned above consider single-time data or mean-time series data; there is a lack of research on combining temporal with spatial information extracted from data. In addition, they suffer from low precision and can only localize the leakage locally due to the uncertainty of the model used and the uncertainty of the measurement data received. The varying mode of daily water demand is indistinguishable from the leak fluctuation mode of pipe networks. How to distinguish exactly which behavior mode the data fluctuation corresponds to is the challenge for leak detection.

The monitoring data of the WDS is a typical time series, and there is a certain spatial correlation between the monitoring devices distributed at different locations of the WDS. After the leakage occurs, multiple monitoring sensors will respond at the same time, producing synchronous pressure changes and showing spatial correlation; besides, the changes will often last for a period of time, and there is autocorrelation in time.

This paper proposes a threshold selection method for leak detection in water distribution systems. It is characterized by (1) fully utilizing temporal varying correlation between the data from multiple pressure sensors; (2) updating threshold values over time; and (3) scanning multiple times for a scanning time window (STW). The main objective is to explore the advantages of data mining based on time series data for leak detection, and to reduce the rate of missing detection and the rate of false alarms for the leakage events effectively. The state of the WDS is changing all the time, and the real-time update of the threshold is beneficial to reduce the influence of the model and measurement uncertainty. Section 2 describes the principle of this methodology in detail. In Section 3, a case study is presented, which uses the water supply network of J City of China to carry out the research on the performance of the leak detection method. Finally, a discussion of the relevant results is given in Section 4.

## 2. Methodology

**Figure 1:**
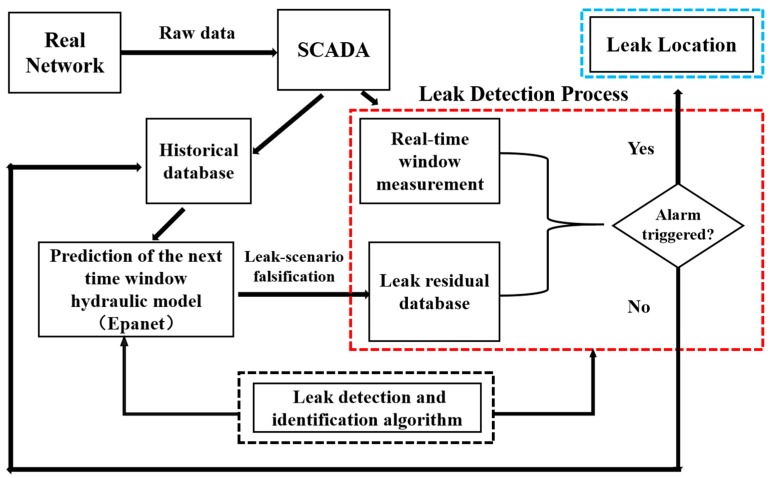
Framework of leak detection and localization methodology based on monitoring data and hydraulic model.

Figure 1 shows the framework of leak detection and localization methodology based on monitoring data and hydraulic model. In this framework, the supervisory control and data acquisition (SCADA) system supplies raw pressure and flow rate data for the prediction and detection process. In the prediction process, it is assumed that such a tool is available to forecast the spatial and temporal demand distribution at the specified time window. Water demand forecasting is based on a combination of pattern recognition and time series models by using the historical water demand database. Then, a simulation is run to obtain the leakage residual database by adding the nodal leakage to normal water demand during the time window. Comparing the measurements from SCADA to the leakage residual database, the leak detection and identification algorithm is used to determine whether the leak occurs. If the detection alarm is not triggered, the network status is classified as a non-fault state, and the prediction hydraulic model of the next specified time window is updated using the current measurement information. Conversely, if the detection alarm is triggered, the next step is to locate the leakage position. The prediction process can be found in many studies [32,33,34]. This paper focuses on the detection process and has the following assumptions: there is only one leak that appears at one time and leaks occurs at a node in the water distribution system model; pressure sensors in the water network are placed in the selected nodes and are working well, and the measured pressures are synthetically generated from the simulated pressure value by adding random noise; the error of the prediction model is considered by adding the noise of the nodal demand to the real nodal demand; sudden special events that may produce significant relevant demand variations are not considered. To achieve the time series threshold estimation process, a few concepts should be recalled here:

- Detection Probability (DP): the proportion of leak events detected in the total number of natural random leak events, and the rate of non-detection (false negative rate) is described as ‘1-DP’.
- Rate of False Alarm (RF): the proportion of false alarms in the total number of natural random events that occur without a leak.

**Figure 2:**
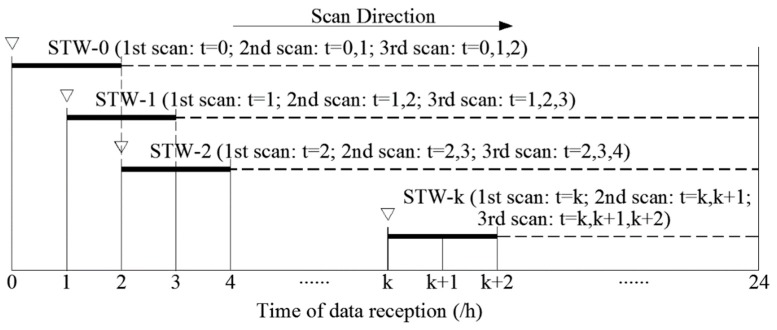
Schematic diagram of real-time leak detection scanning when STWL = 3.

The authors define:

- Scanning Time Window (STW): it is shown in Figure 2. It is a time container covered by continuous time steps. Its length is the number of the covered time step, namely, the scanning time window length (STWL).
- Scanning Process: for STWL of *k*, one complete scan process performs a total of *k* scans. The data is numbered in order of reception time. The *k* scans use the data of time step at *t* = 1, *t* = {1, 2}..., *t* = {1, 2..., *k*}. The scanning process is shown in Figure 2. The RF of the *k*th scan in a STW, RF*_k_*, is assigned to calculate the threshold *C_k_*. A scanning process for an STW requires a corresponding threshold set {*C_k_*}, and the number of elements in the collection is equal to STWL.
- Cumulative Rate of False alarm (CRF): for an STW containing *k* time steps (if STWL = *k*), CRF is the proportion of the total number of false alarms in the total number of natural random events without leakage when a scanning process is completed in that STW.

### 2.1. Leak Scenario Falsification

The core idea of the error-domain model falsification method is to falsify model instances (parameter sets). Then, the maximal plausible errors are determined by combining modeling and measurement uncertainties. There is model uncertainty (*u_model_*) in the numerical model, as well as measurement uncertainty in the actual measured value. As shown in Equation (1), the predicted value (*P_i_*) plus modeling error (*u_model_*) corresponds to the real quantity (*R*), which is equal to the measured value (*y*) plus measurement error (*u_measurement_*) [35,36,37].

(1) $$P_{i}+u_{\mathrm{model}}=R=y+u_{\mathrm{measurement}}$$

The residual difference between the model prediction and the measurement is equal to the difference between the measurement error and the model error by reorganizing the terms of Equation (1). This relation is expressed in Equation (2).

(2) $$P_{i}-y=u_{\mathrm{measurement}}{-u}_{\mathrm{model}}$$

Using the residual of these two uncertainties (representing the expected residual between the predicted and measured values), the corresponding threshold boundaries (by taking the 95% interval of the probability density function) are calculated to evaluate the recognition performance of error-domain model falsification [27].

**Figure 3:**
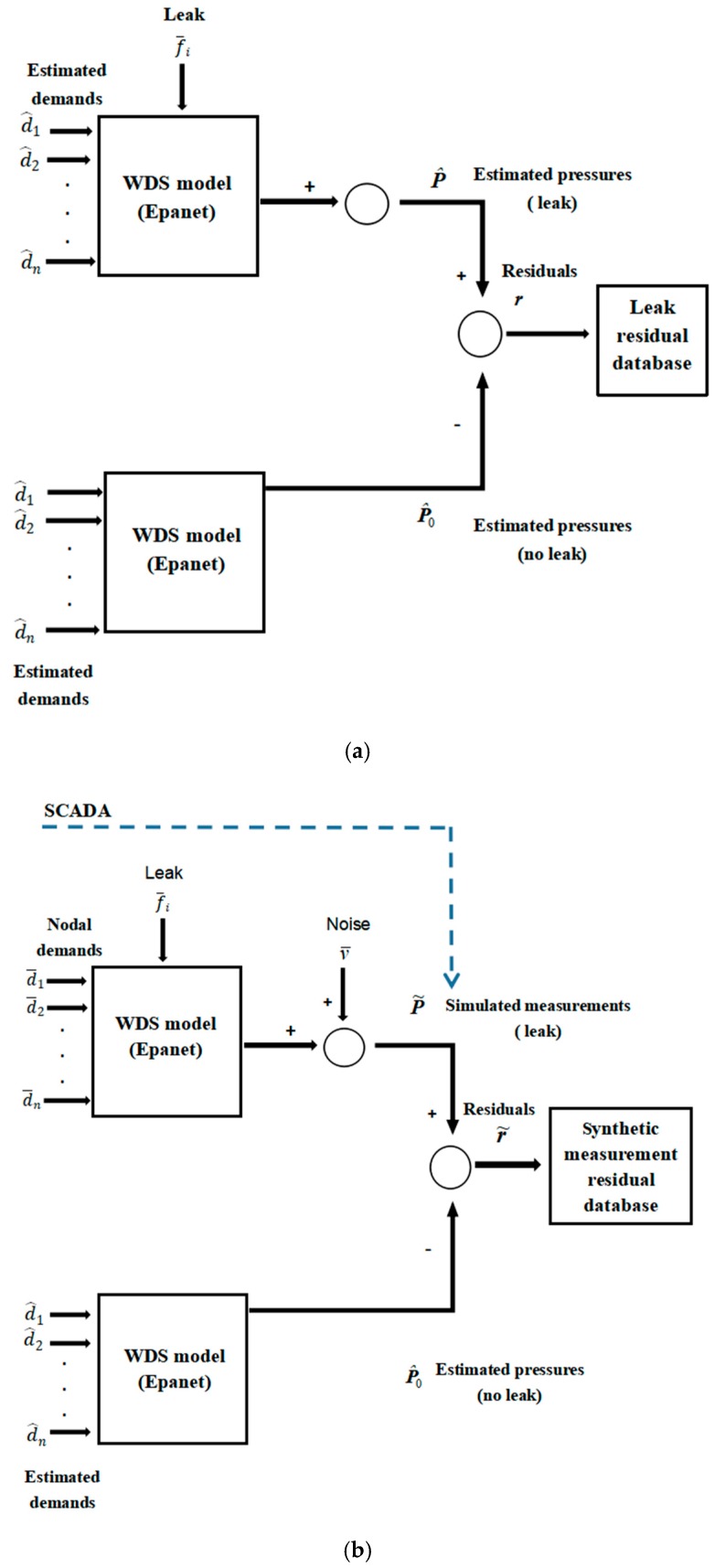
Data generation scheme: (**a**) predicted pressure residual database of the leak scenarios; (**b**) synthetic measurement pressure residual database.

A set of possible leakage scenarios is traversed based on the set of the given parameter values. Each scenario represents one leakage state of the water distribution system (the leakage location and the leakage flow rate are used in this article). It is thought that the scenarios cover the extensive range of possible leakage states of systems, from 20 m^3^/h to 50 m^3^/h with increment of 1 m^3^/h for every node and from 50 m^3^/h to 350 m^3^/h with increment of 5 m^3^/h for every node. As shown in Figure 3a, using the EPANET pressure-driven model to simulate the leak scenario [38], the simulation is a 24-hourly Extended Period Simulation (EPS) based on forecasted water demands. Random noise is added to each node demand value to synthetically generate the node demand value of the predictive model, ${\hat{d}}_{n}={\bar{d}}_{n}+\Delta d$, Δ*d* is the uncertainty of nodal demands. For each leakage scenario, the estimated pressures are calculated where the sensors are placed in the water distribution network. Then, the pressure residual database of the leakage scenarios is obtained by comparing the estimated pressure with leakage to that without leakage. The total number of scenarios in the database equals the number of nodes multiplied by the number of leakage intensities considered.

As shown in Figure 3b, the pressure measurement (simulating raw pressure data supplied by the SCADA system) is synthesized by adding the noise to the simulated pressure value for the leakage scenario or no-leakage scenario. It is compared with the estimated pressure without leakage to obtain the measurement pressure residual. For large quantity leakage scenarios, their pressure residuals form a residual database of the leakage scenario set.

The predicted pressure residual vector ***r*** ($r=\hat{p}-{\hat{p}}_{0}$) of the leakage scenario can be obtained from the leakage residuals database. It characterizes the pressure deviation of all monitoring nodes. The size of the residual vector ***r*** depends on the number of pressure sensors in the network *n_s_*. $\hat{p}$ and ${\hat{p}}_{0}$ are the predicted pressure vector of the nodes, where the pressure sensors are placed under the leakage and no-leakage conditions, respectively.

The measurement pressure residual vector can be obtained by comparing the SCADA data (in this paper, the synthetic pressure is substituted for the real SCADA data) to the estimated pressure without leakage, shown in the residual vector form:

(3) $$\tilde{r}=\tilde{p}-{\hat{p}}_{0}$$

where the size of the residual vector $\tilde{r}$ depends on the number of pressure sensors in the network *n_s_*. $\tilde{p}$ is the pressure vector measured in the nodes where the pressure sensors are placed.

Then, the measurement residual is compared to the leak residual database to calculate their correlation coefficient:

(4) $$C_{r,\tilde{r}}=\frac{cov\left(r,\tilde{r}\right)}{\sqrt{cov\left(r,r\right)cov{\left(\tilde{r},\tilde{r}\right)}^{\prime }}}$$

where $C_{r,\tilde{r}}$ is the correlation coefficient; ***r*** is one of the pressure residual vectors in the leak residual database; $\tilde{r}$ is the measurement residual at time step *t* associated with a potential leakage; $cov\left(r,\tilde{r}\right)=E\left[\left(r-\tilde{r}\right)\left(\tilde{r}-\bar{\tilde{r}}\right)\right]$ is the correlation function between two variables ***r*** and $\tilde{r}$, where $\bar{r}$ = *E*(***r***) and $\bar{\tilde{r}}$ = *E*($\tilde{r}$).

### 2.2. Leak Detection

The larger the correlation coefficient is, the greater the similarity between the current scenario and the leak scenario and the greater the likelihood that the leakage is occurring. The maximum correlation value $C_{r,\tilde{r}max}$ can be used to characterize the most similar leak scenario in the leakage residual database. The maximum correlation is usually used to diagnose the leakage events. When it is less than the threshold value, there is no leak at this time step and, conversely, an alarm is triggered.

Figure 2 shows the schematic diagram of real-time leak detection when the STWL equals 3. From the viewpoint of judging whether a leakage happened at one certain time point, we not only use the current data at that time step to diagnose the leakage (first scan: *t* = *k*), but also wait to get the data at the next time step, combine it with the data at the former time step to diagnose the leakage (second scan: *t* = *k*, *k* + 1), and finally combine all of the data of the continuous three time steps (third scan: *t* = *k*, *k* + 1, *k* + 2). A complete scanning process at each STW performs three scans. After that, the STW moves forward in the direction of time to judge the WDS at the next time step. Leak detection follows a given rule: when the maximum correlation coefficient of all data at the *k*th scan are greater than the *k*th threshold (*C_k_*), it is marked as abnormal condition and an alarm is triggered. If there is no alarm, after the time window moves forward, the qualified data is updated to the historical database to predict the hydraulic model of the next time window. From the viewpoint of the data stream, the data at the current time step is used to diagnose the leakage of WDS at the current time step, *t* = *k*, and the former two time steps, *t* = *k* − 1, and *k* − 2.

### 2.3. Estimation of Threshold Values

The threshold estimation can be calculated by the given RF and the cumulative probability distribution function, if only the data of one time step is used. If more data at continuous time steps is used, the corresponding threshold will be declined by the given RF*_t_* and the cumulative probability distribution function for those continuous time steps, with the CRF value of multiple time steps the same as RF value of one time step.

**Figure 4:**
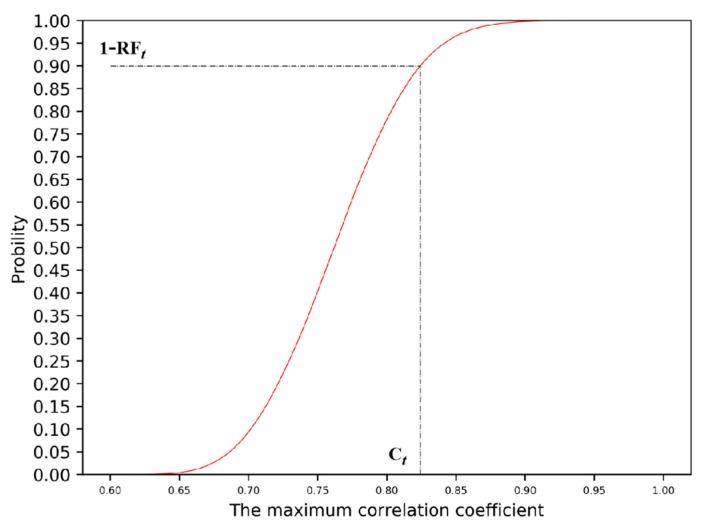
Cumulative probability distribution function of the maximum correlation coefficient.

A large number of measurement results of simulated no-leak scenarios are generated using the Section 2.1 method; the cumulative probability distribution function of maximum correlation coefficient ($C_{r,\tilde{r}max}$) can be obtained by traversing the correlation analysis between the measurement results and the leak residual database. As data is updated over time, the cumulative probability distribution function curve of the maximum correlation coefficient ($C_{r,\tilde{r}max}$) at different time steps can be obtained. A curve of a certain time step is shown in Figure 4. From the cumulative probability distribution, we can get the threshold of $C_{r,\tilde{r}max}$ by given RF*_t_*.

**Figure 5:**
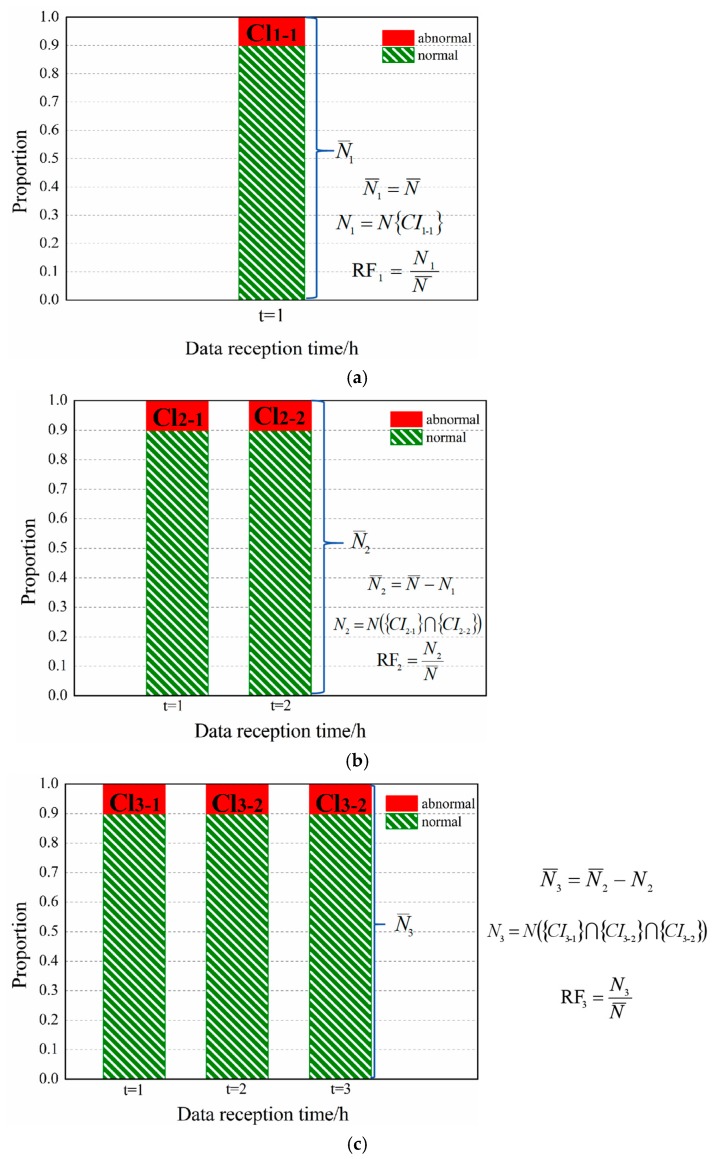
Schematic diagram of estimation of a set of thresholds required for a complete scan when STWL = 3, CRF = 30%: (**a**) first scan; (**b**) second scan; (**c**) third scan.

For single-time data, the corresponding threshold (*C_t_*) can be obtained by assigning RF*_t_* to the cumulative probability distribution function curve. Figure 4 shows the threshold estimation *C_t_* = 0.83 corresponding to RF*_t_* = 10% for single-time data. For continuous time-serial data, Figure 5 gives the schematic diagram of estimation for a set of thresholds {*C_k_*} required for a complete scanning process, taking the STWL = 3 and RF*_t_* = 10% (*t* = 1, 2, 3) as an example. The complete equations and explanation involved in Figure 5 are provided in Appendix A.

The threshold estimation method of the first scan (Figure 5a) in the STW is consistent with the estimation method of the single-time threshold. The second scan (Figure 5b) in the STW uses the threshold *C*_2_ to decide the false alarm events, and the third scan (Figure 5c) uses the threshold (*C*_3_). The threshold (*C*_1_, *C*_2_, and *C*_3_) is given to satisfy the RF*_i_* of different scans in an STW. The sum of the RF*_i_* for the different scans should equal CRF. The average allocation is used in this article (i.e., RF_1_ = RF_2_ = RF_3_).

For an STW with STWL = *k*, according to the assigned rate of false alarm (RF_1_, RF_2_...RF*_k_*) and the cumulative probability distribution function curve of $C_{r,\tilde{r}max}$, the corresponding threshold set {*C_k_*} can be obtained. As the time step advances, each time new time data is received, a scan is performed, and the maximum correlation coefficient at the corresponding time is calculated to obtain a cumulative probability distribution function curve. The rule is that when the maximum correlation coefficients of all data at the *k*th scan are greater than the *k*th scan threshold value (*C_k_*) simultaneously, the alarm is triggered (marked as abnormal). The cumulative rate of false alarm of *k* scans (*t* = 1, *t* = {1, 2}..., *t* = {1, 2..., *k*}) should be equal to the given CRF. (CRF = RF_1_ + RF_2_ + ... + RF*_k_*). Equations (5)–(8) summarizes the formulas involved in *k*th scan:

(5) $$N_{k}=N\left(\left\{CI_{k-1}\right\}\cap \left\{CI_{k-2}\right\}\cdots \left\{CI_{k-k}\right\}\right)$$

(6) $${\bar{N}}_{k}={\bar{N}}_{k-1}-N_{k-1}$$

(7) $$RF_{k}=\frac{N_{k}}{{\bar{N}}_{k}}$$

(8) $$\frac{N\left\{CI_{1-1}\right\}+N\left(\left\{CI_{2-1}\right\}\cap \left\{CI_{2-2}\right\}\right)+\cdots N\left(\left\{CI_{k-1}\right\}\cap \left\{CI_{k-2}\right\}\cdots \left\{CI_{k-k}\right\}\right)}{\bar{N}}=CRF$$

where *CI_k_*_-*k*_ is the set of candidate scenario, the first *k* of the subscript represents the *k*th scan in an STW, and the second one represents the *k*th time data; *N* ({*CI_k_*_-1_}∩{*CI_k_*_-2_}...{*CI_k_*_-k_}) represents the number of intersection elements for candidate scenarios for the data of *k* different time steps; *N_k_* indicates the number of elements marked as abnormal at the *k*th scan; RF*_k_*is the rate of false alarm of the *k*th scan; ${\bar{N}}_{k}$ is the total number of samples for the *k*th scan (the samples identified as abnormal for the *k*th scan will not appear in the total sample of the (*k* + 1)th scan, as shown in Equation (6)); $\bar{N}$ refers to the total number of simulated scenarios, that is, the total number of samples scanned for the first time.

## 3. Case Study

**Figure 6:**
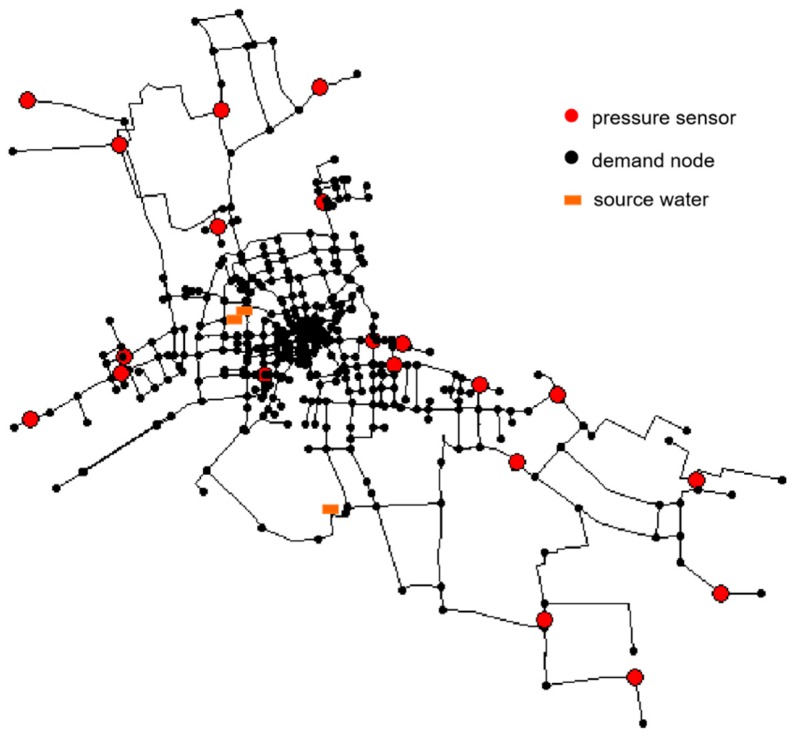
Schematic representation of the water distribution network studied.

The leak detection and location methodology are applied to a real water network model with synthetic data. The network is located in a city in Zhejiang Province, China. It consists of 509 pipes, 491 nodes, and 3 water sources, as shown in Figure 6. A total of 20 pressure sensors (red nodes in Figure 6) are arranged in the network. A model of this network is created using the software EPANET.

### 3.1. Uncertainties

Uncertainty consists of modeling error and measurement error. Modeling errors are caused by model simplification and errors related to model parameters. Model parameters are divided into two groups. The first group contains the main parameters characterizing the leak situation. For leak detection, as mentioned above, these are the leak location and leak strength. Other parameters (referred to as secondary parameters) do not clearly characterize the scenario, mainly including pipe diameter, pipe length, pipe roughness, node elevation, node requirements, and so forth. [27]. The sensitivity studies of these uncertainty values have been conducted to estimate the relative importance of each parameter. The results show that the relative importance of the uncertainty of node demand exceeds 99%.

Random noise is added to each node demand value to synthetically generate the node demand value of the predictive model. Random error follows the standard normal distribution *N* (0, *σ*) where *σ* is the standard deviation of each value, and is set to *σ* = *pμ*/3.27 [39], where *p* is the perturbation ratio, and *μ* is the true nodal demand value. Random noise *N* (0, 0.2 m) is added to the pressure measurement to characterize the measurement errors. Two different data sets are generated with different precision. In data set 1, the disturbance ratio is *p* = 5% for water demand and standard deviation is *σ* = 0.2 m for all pressure measurements. In data set 2, disturbance ratio is *p* = 10% for water demand and standard deviation is *σ* = 0.2 m for all pressure measurements. Thus, set 1 is expected to be more accurate than set 2.

### 3.2. Leak Scenario and Simulated Measurement

The parameter value boundary of the leakage residual database is set as follows: leakage position traverses all nodes (*N_nodes_* = 491), and the leak intensity ranges from 20 m^3^/h to 350 m^3^/h (according to the historical leakage event database compiled by the Water Division, most of the leakage flow rate is concentrated in 20~350 m^3^/h, including small leakage and large burst, and when the leakage flow rate is less than 20 m^3^/h, the pressure fluctuation caused is too small, which is lower than the measurement accuracy of the sensor), with increment of 1 m^3^/h for intervals of 20 m^3^/h to 50 m^3^/h and 5 m^3^/h for intervals of 50 m^3^/h to 350 m^3^/h. The total number of scenarios is equal to the number of nodes multiplied by the number of intensities considered (491 × 91).

For the sample of the simulated leak scenarios, the parameters of the simulated measurement are set as follows: the amount of leakage is divided into six flow rate intervals: 20~30 m^3^/h, 30~40 m^3^/h, 40~50 m^3^/h, 50~100 m^3^/h, 100~200 m^3^/h, 200~350 m^3^/h. A node is selected as the leak position randomly, and a random leakage flow rate in the corresponding interval is selected using a random number generation function in C/C++. For every leakage event, a simulation is run to obtain the pressures at the measurement points. Each sample set corresponds to a scenario where a new leak occurs at time *k*, and the proportion of samples in each flow rate interval of leak is: 10%, 10%, 10%, 20%, 30%, 20%, respectively.

For samples which simulate no-leak scenarios, as described in Section 3.1, two comparison data sets are generated. On the one hand, for evaluating leak detection performance, the number of samples per data set is equal to the number of samples that simulate leak scenarios (this article uses $\bar{N}$ = 10^4^); on the other hand, for threshold estimation, the number of samples is set as 10^5^.

The generation principle of the above three types of samples has been described in Section 2.1 in detail.

### 3.3. Estimation of Threshold Values and Leak Detection

The threshold value used was obtained from the method of Section 2.2 and the synthetic data from Section 3.2 for leak detection testing. The time series is normalized before the data at different times is processed to obtain a threshold, as shown in Equations (9) and (10).

(9) $$R_{\mathrm{nor}(t)}=Max\{C_{r,\tilde{r}\max}\left(1\right),C_{r,\tilde{r}\max}\left(2\right)\cdots C_{r,\tilde{r}\max}\left(N\right)\}$$

(10) $$C_{r,\tilde{r}\max}{\left(N\right)}_{\mathrm{nor}}=\frac{C_{r,\tilde{r}\max}\left(N\right)}{R_{\mathrm{nor}\left(t\right)}}$$

where *R_nor_*_(*t*)_ is the normalization coefficient at time t; *N* represents the total number of samples; $C_{r,\tilde{r}\max}\left(N\right)$ is maximum correlation coefficient of the *N*th sample at time *t*; $C_{r,\tilde{r}\max}{\left(N\right)}_{\mathrm{nor}}$ indicates the maximum correlation coefficient of the *N*th sample after normalization.

The online monitoring *k*-hour scenario is simulated, and then the corresponding *k*-group thresholds are obtained for a given STWL of 1~24 by threshold analysis. Table 1 shows threshold set for each STW with entire time horizon obtained by threshold analysis when CRF = 10%, STWL = 3. The threshold set corresponding to the scanning process varies over time.

## 4. Results and Discussion

**Figure 7:**
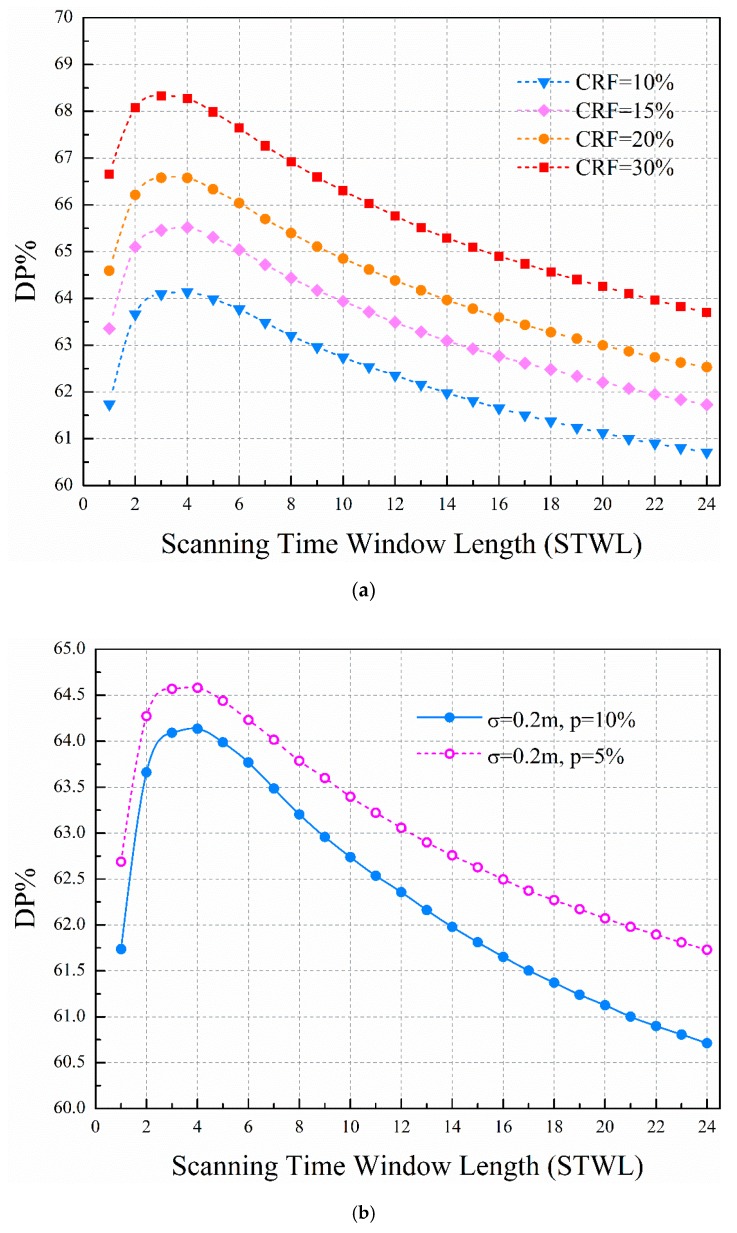
(**a**) Leakage detection probability for different given CRF when σ = 0.2 m, p = 10%; (**b**) leakage detection probability for different tested data sets when CRF = 10%.

Two different predicted water demand errors (*p* = 5% and 10%) are considered. The cumulative rate of false alarm (CRF) is set from 5% to 30%, with an increment of 2.5%. The RF*_k_* is set to average allocation in this paper. Noticeably, it has been confirmed that there is a better allocation ratio scheme (see the Appendix B for details); it is necessary to automatically optimize the allocation ratio by using optimization methods in the future. For every CRF, the DP versus STWL curves are obtained. The curves shown in Figure 7a are the scenarios of CRF = 10%, 15%, 20%, and 30%, with leak intensity ranges from 20 m^3^/h to 350 m^3^/h. Compared to the single-time STW, we can clearly see that the proposed method using multitime STW can effectively improve the detection probability for a given cumulative rate of false alarm.

The detection probability increases with the STWL and then descends with the STWL. In the initial STWLs, when the STWL is set from 1 to 4 (the time step of one hour is used in this paper), the detection probability is significantly improved. After that, the detection probability shows a downward trend with the increasing STWL. There is an optimal range of STWL for detection probability. A wide STW means a large number of scans in an STW. The last number of scans cover continuous monitoring data over the STW. For successful leakage detection, all correlation coefficients of the data should support the same judgments. However, the data includes random demand noise and pressure measurement noise. The longer the data series is used, the harder it is to make consistent judgment. Thus, the detection probability descends with the increase of the STWL when the STWL is over a certain value.

In comparison with the clustering-based method using cosine distance to evaluate dissimilarity between data from multiple flow sensors to detect bursts [40], we can find a similar trend where RF increases with the decrease in window size when the DP remains constant, although different analysis methods are used for multitime series data. Using the optimal STWL value can enhance DP by 3%, compared to using single-time STW (STWL = 1) for leak detection. When STWL increases beyond 16, the detection probability is lower than that of single-time STW. The discussion of the influence of STWL on detection performance can be found in many other leak detection methods. Abokifa et al. [41] demonstrated that an optimal window period can be selected to achieve the best performance, while smaller and larger windows would generally yield less accurate results. Wu et al. [42] drew the conclusion that detection performance of the method changes only slightly when STWL varies from 1 to 6 days.

Figure 7b shows that the predicted water demand error has an obvious effect on the proposed leakage detection method. The water demand disturbance of set 1 (*p* = 5%) is smaller than for set 2 (*p* = 10%), and the leakage detection probability of set 1 is higher than for set 2. On the other hand, the leakage detection probability shows a similar trend for different cumulative rates of false alarm between set 1 and set 2. The higher the cumulative rate of false alarm, the higher the leakage detection probability. Eliminating model errors improves leakage detection probability.

**Figure 8:**
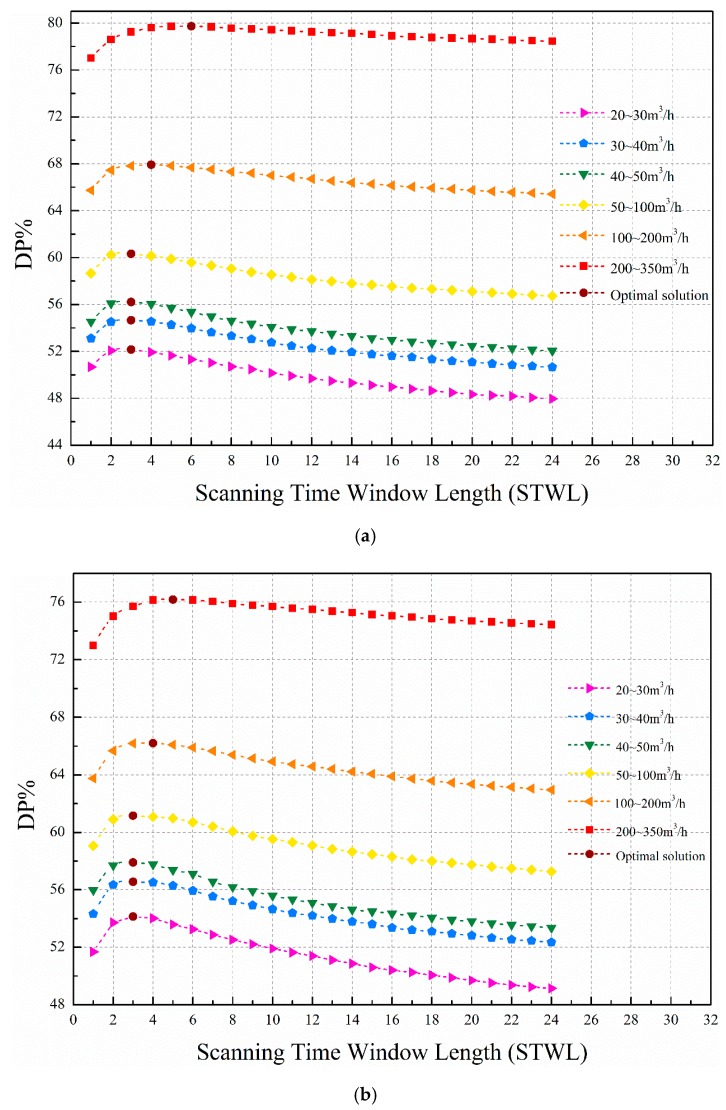
Leakage detection probability for different flow rate intervals when CRF = 10%: (**a**) σ = 0.2 m, *p* = 5%; (**b**) σ = 0.2m, *p* = 10%.

In order to exploit the advantages of the proposed method using multitime STW, the DP versus STWL curves for different leakage flow rate intervals are shown in Figure 8 when the CRF is set at 10%. Six intervals are divided: 20~30 m^3^/h, 30~40 m^3^/h, 40~50 m^3^/h, 50~100 m^3^/h, 100~200 m^3^/h, 200~350 m^3^/h. The optimal solution points indicate the optimal STWL value corresponding to the highest detection probability within the corresponding flow rate interval. The curves of each flow rate interval show a similar trend for differently tested data sets. The detection performance of the large flow rate interval is better than that of the small flow rate interval. This is primarily due to the relationship between the fluctuation caused by the leakage and the degree of uncertainty. Small leakages located in areas with high uncertainty are not detectable due to the small variations caused by them; the fluctuation caused by the leakage is smaller than the fluctuation of the uncertainty of the affected node and may be overlooked. In short, fluctuation caused by larger leakages is greater and less likely to be masked under uncertainty at a given level. The STWLs that have optimal detection performance for different flow rate intervals are concentrated from 3 to 6 for the tested data set. Similarly, Wu et al. [40] chose the STWL of approximately 6 h for burst detection. However, in his paper, every STW only scans once, while a total of *k* scans (*t* = 1, *t* = {1,2}...,*t* = {1,2,...,*k*}) are performed for every STW in this paper.

In comparison with the single-time model-based leak detection methodology [24], the method in this paper fully uses the temporal varying (multiple time rather than single time) correlation between the data from multiple pressure sensors, and the detection performance is improved. In addition, Casillas Ponce et al. [43] proposed a model-based approach for leak detection and location, which considers an extended time horizon analysis of pressure sensitivities. To extract data information in the time horizon, the authors look for the mean value or mode in an STW for different metrics (binarization, correlation, angle between vectors, and Euclidean distance). The advantage of the method using the correlation coefficient metric in this paper is that multitime series residual analysis can be more sensitive to leakage than using mode or mean value in an STW.

**Figure 9:**
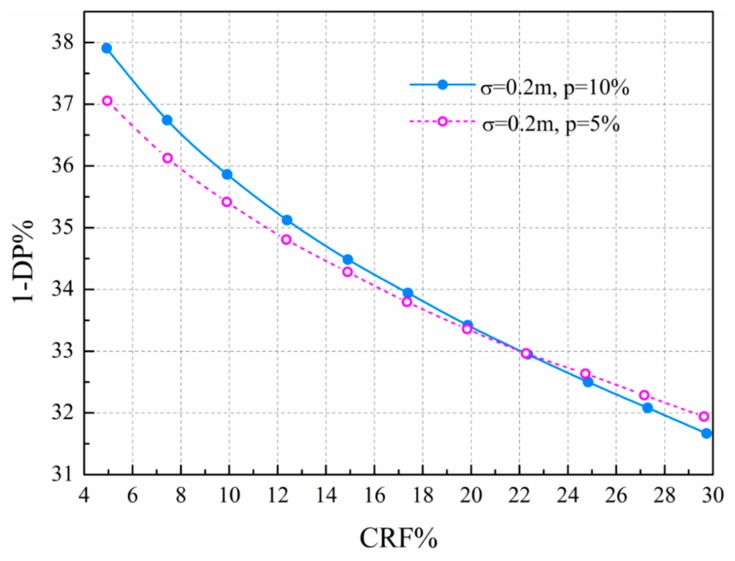
Relationship between cumulative rate of false alarm and rate of missing detection. (Each STWL was tested for 24 different start times, and the vertical coordinate value of the point in Figure 7 and Figure 8 is the average of 24 different DPs. Figure 9 utilizes a similar processing method to obtain an average of 24 1-DPs when selecting the optimal solution of STWL.)

Figure 9 shows the trade-off curve for the given cumulative rate of false alarm and rate of missing detection of the tested data set when selecting the optimal solution of STWL. The users can make the decisions to choose a combination between the false positive rate (rate of false alarm) and false negative rate (rate of missing detection) according to the actual situation, and every point of the curve corresponds to the solution of the optimal STWL. For the leakage detection performance at the optimal STWL, the demand error of *p* = 10% decreased the DP of 1% more than that of *p* = 5%.

## 5. Conclusions

This work proposes a leak detection method combined with multitime series. Leak detection is based on the comparison between real-time online data (simulated measurements) and predictive model data. The proposed method is about threshold selection method based on time series monitoring data. The detection is based on multitime series residual analysis. The influences of the uncertainty of the prediction model and measurement noise are considered for this proposed method.

The higher the CRF, the higher the leakage detection probability. Under given parameters, detection performance for the large leakage is better than for the small leakage because the pressure change caused by the low-intensity leakage may be overlooked by the modeling and measurement uncertainties. For the optimal STWL, the DP with demand error of p = 10% decreased by 1% over p = 5%. The future improvement of the water demand prediction method and installing of extra sensors can reduce the effects of uncertainty. For different flow rate intervals and different cumulative rate of false alarm, the leakage detection performance and the STWL show a positive correlation trend firstly, and then the trend is a negative correlation. The STWLs for achieving optimal detection performance for different conditions are concentrated from 3 to 6 for the tested data set. Using the optimal STWL can enhance DP of 3% more than using single-time STW for leak detection.

The multitime series analysis method is used to enhance the detection probability and to reduce the rate of missing detection and the rate of false alarm effectively, compared with the single-time series method. Many factors (the STWL, RF_k_, CRF, ∆d, etc.) will affect the performance of the detection method. The diversity combination of the parameter settings will be the next research work. The main purpose of this paper is to prove the feasibility of the method. In addition, various leakage indicators can be obtained by different metrics, and then leakage detection methods derived can be coupled with this method, which has wide applicability. Future work will consider the minimum detectable leak depending on the resolution of sensors. In addition, flow sensors will be tested and compared with pressure sensors in order to assess which is the best option. A real-case test will be performed when real data become available in the future. Finally, the applicability of the method will be evaluated by comparison with other methods.

## Figures and Tables

**Table 1:** Threshold set of the *k*th scan window when STWL = 3, CRF = 10%.

Time of Data Reception (/h)	*C*_1_	*C*_2_	*C*_3_
1	0.8505	0.8096	0.79414
2	0.86942	0.83346	0.81404
3	0.89819	0.85094	0.83715
4	0.89095	0.86504	0.84344
5	0.92704	0.87481	0.84578
6	0.8965	0.85363	0.83573
7	0.8902	0.85424	0.83336
8	0.9067	0.86027	0.84362
9	0.89703	0.86366	0.85382
10	0.91036	0.89074	0.85721
11	0.93878	0.88023	0.84261
12	0.89223	0.844	0.81992
13	0.88657	0.84464	0.82792
14	0.88874	0.85232	0.8236
15	0.90972	0.85166	0.83497
16	0.88232	0.85288	0.83348
17	0.90894	0.86886	0.84315
18	0.90221	0.8549	0.81224
19	0.8947	0.82221	0.80851
20	0.85178	0.83041	0.80646
21	0.91813	0.86093	0.84623
22	0.87678	0.85378	0.8401
23	0.92034	0.88474	0.83387
24	0.91538	0.83861	0.80631

**Figure A1:**
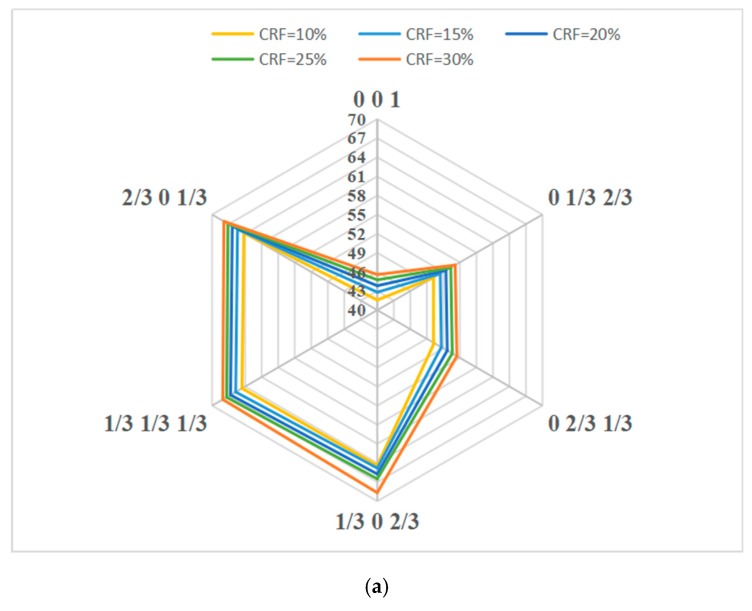

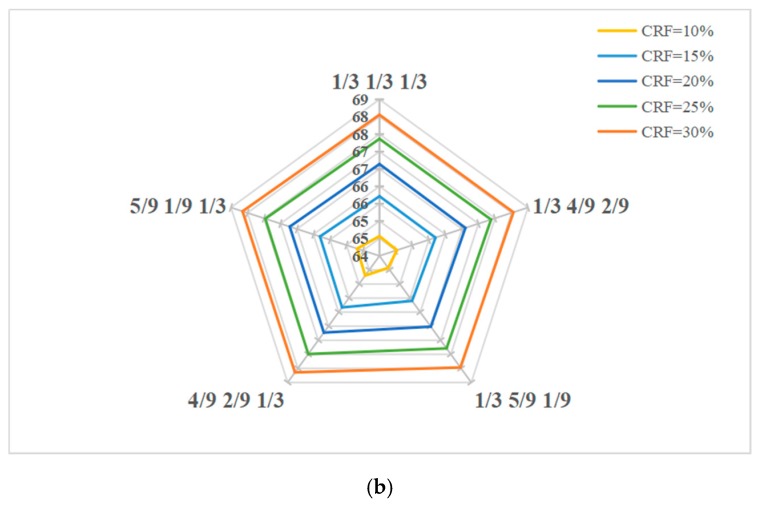
The DPs of different allocation ratio schemes for different given CRF when STWL = 3: (**a**) σ = 0.2m, *p* = 10%; (**b**) σ = 0.2m, *p* = 5%.

## Appendix A

The equations involved in Figure 5 in this study are summarized as follows:

(A1) $${\bar{N}}_{1}={\bar{N}}_{}$$

(A2) $$P\left(C_{r,\tilde{r}\max1}\geq C_{1}\right)*\bar{N}=N\left\{CI_{1-1}\right\}$$

(A3) $$N_{1}=N\left\{CI_{1-1}\right\}$$

(A4) $${\mathrm{RF}}_{1}=\frac{N_{1}}{{\bar{N}}_{}}$$

where ${\bar{N}}_{1}$ is the total number of samples for the 1st scan; $P\left(C_{r,\tilde{r}\max1}\geq C_{1}\right)$ is the probability that the maximum correlation coefficient of the first time is greater than the threshold value (*C*_1_); $\bar{N}$ refers to the total number of simulated scenarios; *CI*_1-1_ is the set of candidate scenario indexes representing the first scan threshold (the former 1) for the first time data (the latter 1); *N*_1_ indicates the number of elements marked as abnormal at the first scan; RF_1_ is the rate of false alarm of the first scan.

(A5) $${\bar{N}}_{2}=\bar{N}-N_{1}$$

(A6) $$P\left(C_{r,\tilde{r}\max1}\geq C_{2}\right)*{\bar{N}}_{2}=N\left\{CI_{2-1}\right\}$$

(A7) $$P\left(C_{r,\tilde{r}\max2}\geq C_{2}\right)*{\bar{N}}_{2}=N\left\{CI_{2-2}\right\}$$

(A8) $$N_{2}=N\left(\left\{CI_{2-1}\right\}\cap \left\{CI_{2-2}\right\}\right)$$

(A9) $${\mathrm{RF}}_{2}=\frac{N_{2}}{{\bar{N}}_{}}$$

where ${\bar{N}}_{2}$ is the total number of samples for the second scan; $P\left(C_{r,\tilde{r}\max1}\geq C_{2}\right)$, $P\left(C_{r,\tilde{r}\max2}\geq C_{2}\right)$ are the probability that the maximum correlation coefficient of the first time and second time are greater than the threshold value, respectively, when the threshold value is *C*_2_; *CI*_2-1_, *CI*_2-2_ are the set of candidate scenario indexes representing the second scan threshold for the first time data and second time data, respectively; *N*_2_ indicates the number of elements marked as abnormal at the second scan; RF_2_ is the rate of false alarm of the second scan.

(A10) $${\bar{N}}_{3}={\bar{N}}_{2}-N_{2}$$

(A11) $$P\left(C_{r,\tilde{r}\max1}\geq C_{3}\right)*{\bar{N}}_{3}=N\left\{CI_{3-1}\right\}$$

(A12) $$P\left(C_{r,\tilde{r}\max2}\geq C_{3}\right)*{\bar{N}}_{3}=N\left\{CI_{3-2}\right\}$$

(A13) $$P\left(C_{r,\tilde{r}\max3}\geq C_{3}\right)*{\bar{N}}_{3}=N\left\{CI_{3-3}\right\}$$

(A14) $$N_{3}=N\left(\left\{CI_{3-1}\right\}\cap \left\{CI_{3-2}\right\}\cap \left\{CI_{3-2}\right\}\right)$$

(A15) $${\mathrm{RF}}_{3}=\frac{N_{3}}{{\bar{N}}_{}}$$

where ${\bar{N}}_{3}$ is the total number of samples for the third scan; $P\left(C_{r,\tilde{r}\max1}\geq C_{3}\right)$, $P\left(C_{r,\tilde{r}\max2}\geq C_{3}\right)$, $P\left(C_{r,\tilde{r}\max3}\geq C_{3}\right)$ are the probability that the maximum correlation coefficient of the first time, second time, and third time are greater than the threshold value, respectively, when the threshold value is *C*_3_; *CI*_3-1_, *CI*_3-2_, *CI*_3-3_ are the set of candidate scenario indexes representing the third scan threshold for the first time data, second time data, and third time data, respectively; *N*_3_ indicates the number of elements marked as abnormal at the third scan; RF_3_ is the rate of false alarm of the third scan.

RF_1_ = RF_2_ = RF_3_ (A16)

CRF = RF_1_+RF_2_+RF_3_ = 30% (A17)

## Appendix B

Results of different allocation ratio schemes are shown as follows:

The category axis of the radar chart represents the different schemes of the allocation ratio (e.g., ‘1/3 1/3 1/3’ represents RF_1_ = RF_2_ = RF_3_ = CRF/3), and the value axis represents the corresponding DP. Partial allocation ratio schemes are shown in Figure A1 for different conditions.
